# Knowledge graph-derived feed efficiency analysis via pig gut microbiota

**DOI:** 10.1038/s41598-024-64835-6

**Published:** 2024-06-17

**Authors:** Junmei Zhang, Qin Jiang, Zhihong Du, Yilin Geng, Yuren Hu, Qichang Tong, Yunfeng Song, Hong-Yu Zhang, Xianghua Yan, Zaiwen Feng

**Affiliations:** 1 National Key Laboratory of Agricultural Microbiology, College of Informatics, College of Animal Sciences and Technology, College of Veterinary Medicine, Huazhong Agricultural University, Wuhan, 430070 China; 2 Yazhouwan National Laboratory (YNL), Sanya, 572025 China

**Keywords:** Pig, Gut microbiota, Knowledge graph, Feed efficiency, Computational biology and bioinformatics, Microbiology

## Abstract

Feed efficiency (FE) is essential for pig production, has been reported to be partially explained by gut microbiota. Despite an extensive body of research literature to this topic, studies regarding the regulation of feed efficiency by gut microbiota remain fragmented and mostly confined to disorganized or semi-structured unrestricted texts. Meanwhile, structured databases for microbiota analysis are available, yet they often lack a comprehensive understanding of the associated biological processes. Therefore, we have devised an approach to construct a comprehensive knowledge graph by combining unstructured textual intelligence with structured database information and applied it to investigate the relationship between pig gut microbes and FE. Firstly, we created the *pgmReading* knowledge base and the domain ontology of pig gut microbiota by annotating, extracting, and integrating semantic information from 157 scientific publications. Secondly, we created the *pgmPubtator* by utilizing PubTator to expand the semantic information related to microbiota. Thirdly, we created the *pgmDatabase* by mapping and combining the ADDAGMA, gutMGene, and KEGG databases based on the ontology. These three knowledge bases were integrated to form the Pig Gut Microbial Knowledge Graph (PGMKG). Additionally, we created five biological query cases to validate the performance of PGMKG. These cases not only allow us to identify microbes with the most significant impact on FE but also provide insights into the metabolites produced by these microbes and the associated metabolic pathways. This study introduces PGMKG, mapping key microbes in pig feed efficiency and guiding microbiota-targeted optimization.

## Introduction

The gut microbiota of pigs plays a pivotal role in nutrient digestion and absorption and profoundly influences the host's health, which is affected by many factors including genetics, diet, disease, and rearing environment^1^. Feed efficiency (FE) is one of the key performances in pig production, is intricately linked with the gut microbiota's role in nutrient digestion, implying its potential influence on FE^2^. Therefore, identifying gut microbial taxa associated with FE can offer valuable insights to improve the profitability and sustainability of the pig industry.

Many studies have explored the relationship between feed efficiency and gut microbiota in pigs^3–8^. Comprehensive literature reviews and meta-analyses have been the favored methods to gain a systematic perspective on this relationship^2,9^. However, with the rapid expansion of publications, it's becoming increasingly challenging for researchers to stay updated without significant effort. In response, recent advancements have pivoted to text-mining techniques to extract and identify non-explicit relationships between concepts in literature-based research^10^, with an increasing adoption of knowledge engineering techniques for relational discovery.

Knowledge graph technology is a pivotal tool in text-mining and semantic web methods. It is a standardized integration and analysis technology for big data that utilizes a standardized conceptual model, ontological terminology, and syntactic format to model and describe data. It also enables the description of knowledge and the modeling of associative relationships between entities in the world through graphical models^11^. Big data management based on knowledge graph technology has the advantages of standardized expression, high correlation, and strong ability to be mined in depth, which can effectively query, discover and infer complex relationships between things and concepts from big data^12,13^, and has become an important paradigm for big data integration and analysis in many research fields of life sciences^14^, including intelligent retrieval of big data in agriculture and biology^15–18^, precision medical treatment^19^, intelligent bio-breeding^20,21^, drug screening^22^, microbial colony-disease prediction^23,24^, and diagnosis of crop diseases and pests^13^. However, the application of knowledge map in pig gut microbiota and feed efficiency is still in the preliminary research stage.

In this study, we proposed a novel knowledge graph which we named as PGMKG to identify and summarize the relationships between gut microbiota and pig feed efficiency. We aim to construct a specialized ontology within this domain, create a knowledge graph of pig gut microbiota, and identify key microbes that are closely related to the improvement of feed efficiency of pigs, thus assisting researchers in optimizing decisions when attempting to optimize feed efficiency through microbiota-targeted strategies.

## Results

### The domain ontology of pig gut microbiota

**Figure 1:**
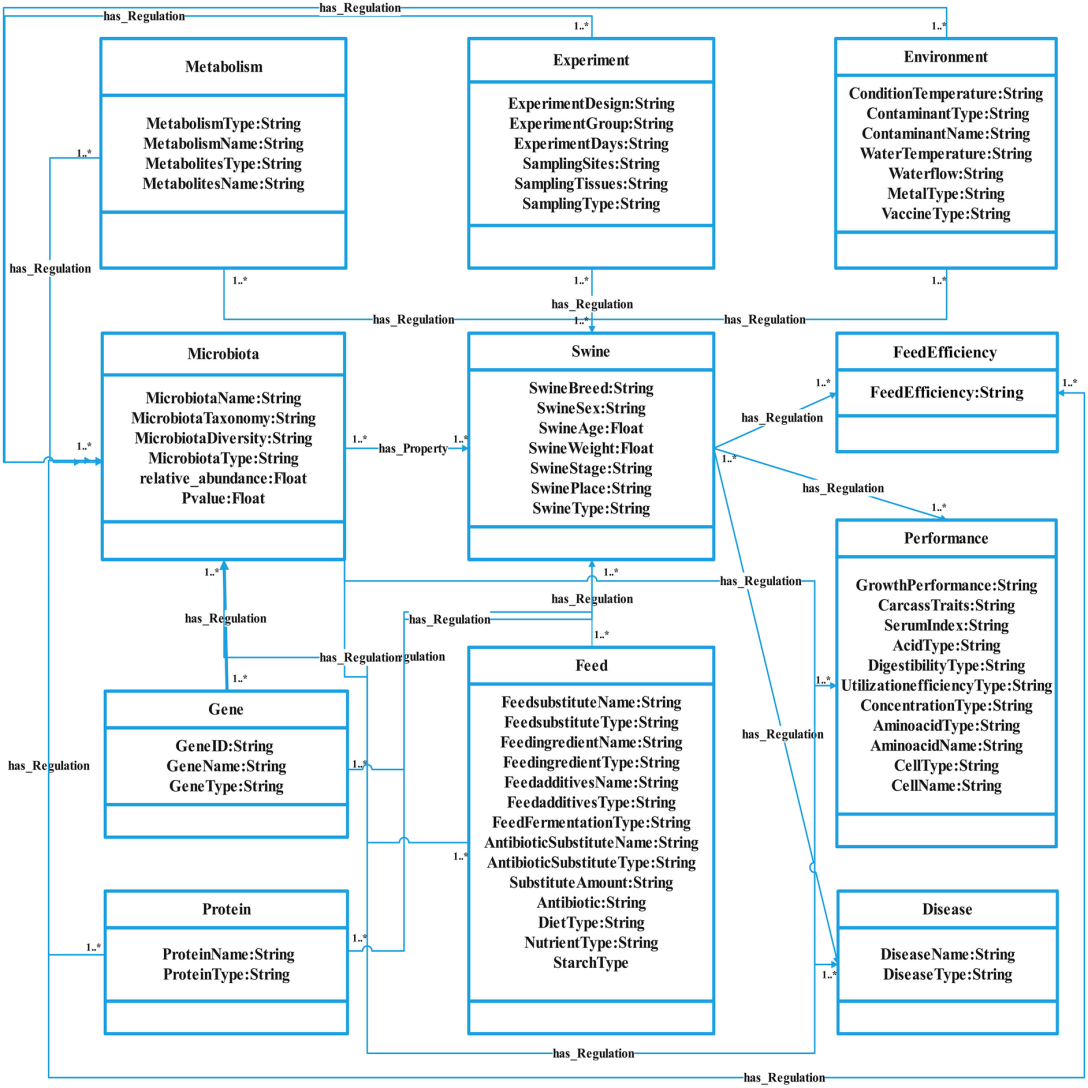
The ontology of pig gut microbiota domain. Each box represents an entity type, and the connecting line in the middle indicates the relationship between the two.

To accurately grasp and express domain knowledge, the domain ontology of pig gut microbes in this study was established based on the scientific literature related to pig gut microbes and feed efficiency (Fig. 1), which defined entity categories, attributes, and relationship types, providing a shared framework for the semantic relationships among the data. This ontology comprises 11 classes that describe the basic information about pigs, including the feed, metabolites, gut microbes, growth performance, and feed efficiency. Among them, basic information of pigs includes their breed, gender, weight, growth stage and type; gut microbial information includes its name, taxonomy, diversity, type and other related details; experimental information includes experimental design, grouping, duration, sampling position and sampling type. Feed information includes feed additives, feed substitutes, feed ferments, types and names of antibiotics, etc. Growth performance information included carcass traits, serum indicators, digestibility, energy utilization, etc. In addition, the ontology also includes other information such as proteins, genes, metabolic pathways. The ontology can clearly represent the domain knowledge of pig gut microbes and their relationships. It is the premise of big data management and application of pig gut microbes, and provides the basis for deep learning and mining.

### Concepts and triplet statistics in the knowledge graph

To integrate datasets from different sources, we standardized and integrated various data through processes such as data collection, data cleaning, entity relationship identification, graph construction and storage, and visualization. The *pgmReading* based on manual screening reading has a total of 2307 entities and 6217 triples. The *pgmPubtator* based on tool automatic construction contains 26,203 nodes and 23,948 triples. The *pgmDatabase* based on the data related to the pig gut microbial database generates 14,297 nodes and 28,731 triples. A total of 42,547 nodes and 58,896 triples could be inferred from the combination of these three datasets (Table 1).

**Table 1:** Number of triples in knowledge bases.

Knowledge base	Node	Triple
pgmReading	2307	6217
pgmPubtator	26,203	23,948
pgmDatabase	14,297	28,731
PGMKG = pgmReading + pgmPubtator + pgmDatabase	42,547	58,896

### Gut microbiota associated with feed efficiency

To demonstrate the primary query functionality of our knowledge graph, we queried gut microbiota related to feed efficiency. From the data gleaned from the *pgmReading* knowledge base, it was observed that that *Ruminococcus flavefaciens*, *Anaerostipes*, *Bacteroidaceae*, *Bacteroides, Bifidobacterium*, *Blautia*, *Campylobacter*, *Cellulosilyticum*, *Christensenellaceae, Clostridiaceae_1*, *Coriobacteriaceae*, *Lachnospiraceae*, *Leeia*, *Lentisphaerae, Methanobrevibacter*, *Mucispirillum*, *Prevotella*, *Prevotella 9*, *Prevotellaceae TCG-001*, *Rothia*, *Ruminococcaceae*, *Subdoligranulu*, *Treponema*, *Bacteroidales*, *Clostridiales*, *Colinsella*, *Lactobacillus* and *Paraprevotella clara* were positively correlated with feed efficiency. Among them, *Bacteroidales*, *Bifidobacterium*, *Clostridiales*, *Colinsella*, *Lactobacillus*, *Paraprevotella clara* and *Prevotella copri* were significantly positively associated with feed efficiency (*P* < 0.05).

**Figure 2:**
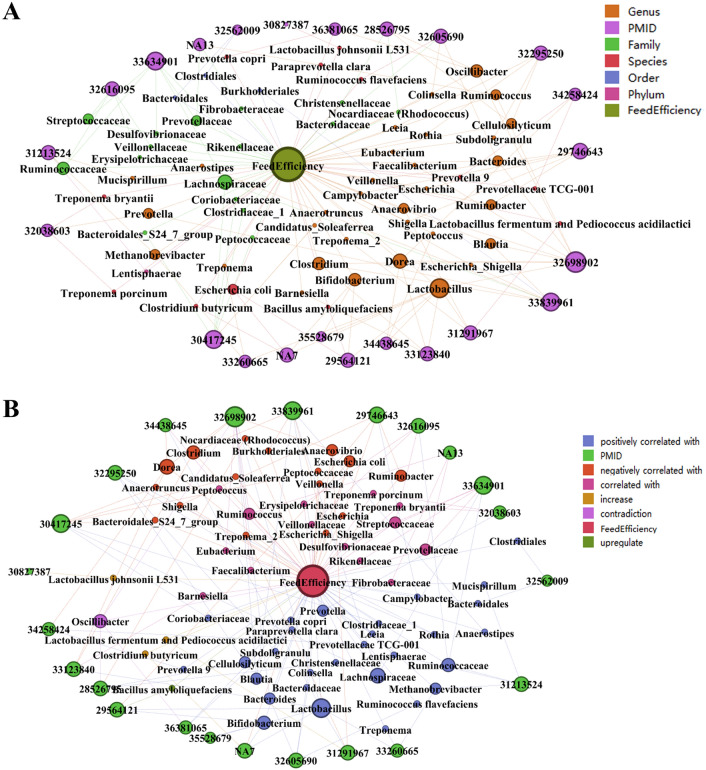
All microbiota related to feed efficiency in *pgmReading*. (**A**) Each node represents an entity, different colours represent different entity types, and the node size represents the number of connecting line; in other words, the larger the node, if there is more literature studying the microbe. (**B**) In order to show the relationship between microorganisms and FE, we use different node colours to represent the relationship between them. The legend in the upper right corner shows the meanings of different colors. The relationships between feed efficiency and microorganisms shown in the figure include positively correlated with, negatively correlated with, correlated with, increase, upregulate, etc. Obviously, the number of microorganisms positively correlated with FE is the largest in the graph (25 microbes).

Furthermore, the microbes negatively correlated with FE are as follows: *Anaerotruncus*, *Anaerovibrio*, *Bacteroidales_S24_7_group*, *Burkholderiales*, *Candidatus_Soleaferrea*, *Clostridium*, *Dorea*, *Escherichia*, *Escherichia-Shigella*, *Nocardiaceae (Rhodococcus)*, *Peptococcaceae*, *Ruminobacter*, *Shigella*, *Treponema_2*, *Veillonella* and *Escherichia coli*, among which *Escherichia coli* and *Ocilibacter* were significantly negatively associated (*P* < 0.05). *Clostridium butyricum*, *Lactobacillus johnsonii L531* and* “*combined *Lactobacillus fermentum* and *Pediococcus acidilactici”* have protential roles in improving FE. Otherwise, *Bacillus amyloliquefaciens* can significantly improve FE. Notably, *Lactobacillus* is the most frequently mentioned as a key microbe in our knowledge base, suggesting its central role in FE regulation, followed by *Dorea* and *Lachnospiraceae* (Fig. 2 and Table 2). The literature source of each microbe is also provided in Fig. 2, which can be used by interested researchers. In view of the query results of *pgmReading* and *pgmPubtator*, we can further confirm that *Lactobacillus* is the most frequently studied microbes in literature related to feed efficiency, followed by *Bifidobacterium*, with *Bacteroides* ranking third (Table S1).

**Table 2:** All microbiota related to feed efficiency in *pgmReading*.

Microbiota taxonomy	PMID	Microbiota name	Relation	Feed efficiency
Genus	33839961	*Anaerostipes*	Positively_correlated_with	Feed efficiency
Genus	30417245	*Anaerotruncus*	Negatively_correlated_with	Feed efficiency
Genus	31291967	*Anaerovibrio*	Negatively_correlated_with	Feed efficiency
Genus	32698902		Significantly_correlated	Feed efficiency
Species	29564121	*Bacillus amyloliquefaciens*	Upregulate	Feed efficiency
Family	32605690	*Bacteroidaceae*	Positively_correlated_with	Feed efficiency
Order	32562009	*Bacteroidales*	Significantly_positively_correlated	Feed efficiency
Family	30417245	*Bacteroidales_S24_7_group*	Negatively_correlated_with	Feed efficiency
Genus	32698902	*Bacteroides*	Significantly_correlated	Feed efficiency
Genus	32605690		Positively_correlated_with	Feed efficiency
Genus	33123840	*Barnesiella*	Significantly_correlated	Feed efficiency
Genus	35528679	*Bifidobacterium*	Positively_correlated_with	Feed efficiency
Genus	29564121		Significantly_positively_correlated	Feed efficiency
Genus	7		Positively_correlated_with	Feed efficiency
Genus	32698902	*Blautia*	Significantly_correlated	Feed efficiency
Genus	33839961		Positively_correlated_with	Feed efficiency
Order	33839961	*Burkholderiales*	Negatively_correlated_with	Feed efficiency
Genus	29746643	*Campylobacter*	Positively_correlated_with	Feed efficiency
Genus	30417245	*Candidatus_Soleaferrea*	Negatively_correlated_with	Feed efficiency
Genus	32295250	*Cellulosilyticum*	Positively_correlated_with	Feed efficiency
Genus	28526795			Feed efficiency
Family	28526795	*Christensenellaceae*	Positively_correlated_with	Feed efficiency
Family	30417245	*Clostridiaceae_1*	Positively_correlated_with	Feed efficiency
Order	32562009	*Clostridiales*	Significantly_positively_correlated	Feed efficiency
Genus	32698902	*Clostridium*	Significantly_correlated	Feed efficiency
Genus	33123840		Significantly_correlated	Feed efficiency
Genus	33839961		Negatively_correlated_with	Feed efficiency
Species	7	*Clostridium butyricum*	Increase	Feed efficiency
Genus	32605690	*Colinsella*	Significantly_positively_correlated	Feed efficiency
Family	30417245	*Coriobacteriaceae*	Positively_correlated_with	Feed efficiency
Family	33634901	*Desulfovibrionaceae*	Significantly_correlated	Feed efficiency
Genus	32698902	*Dorea*	Significantly_correlated	Feed efficiency
Genus	33123840		Significantly_correlated	Feed efficiency
Genus	34438645		Significantly_correlated	Feed efficiency
Genus	34258424		Negatively_correlated_with	Feed efficiency
Family	32616095	*Erysipelotrichaceae*	Correlated_with	Feed efficiency
Genus	29746643	*Escherichia*	Negatively_correlated_with	Feed efficiency
Species	29564121	*Escherichia coli*	Significantly_negatively_correlated	Feed efficiency
Species	7		Negatively_correlated_with	Feed efficiency
Genus	33839961	*Escherichia–Shigella*	Negatively_correlated_with	Feed efficiency
Genus	32698902	*Eubacterium*	Significantly_correlated	Feed efficiency
Genus	32698902	*Faecalibacterium*	Significantly_correlated	Feed efficiency
Family	13	*Fibrobacteraceae*	Significantly_correlated	Feed efficiency
Family	33634901	*Lachnospiraceae*	Significantly_correlated	Feed efficiency
Family	30417245		Positively_correlated_with	Feed efficiency
Family	33839961		Positively_correlated_with	Feed efficiency
Family	33634901		Significantly_correlated	Feed efficiency
Genus	31291967	*Lactobacillus*	Positively_correlated_with	Feed efficiency
Genus	32698902		Significantly_correlated	Feed efficiency
Genus	33260665		Positively_correlated_with	Feed efficiency
Genus	33123840		Significantly_correlated	Feed efficiency
Genus	35528679		Positively_correlated_with	Feed efficiency
Genus	29564121		Significantly_positively_correlated	Feed efficiency
Genus	7		Positively_correlated_with	Feed efficiency
Genus	34438645		Significantly_correlated	Feed efficiency
Species	31291967	*Lactobacillus fermentum and Pediococcus acidilactici*	Increase	Feed efficiency
Species	30827387	*Lactobacillus johnsonii L531*	Increase	Feed efficiency
Genus	32295250	*Leeia*	Positively_correlated_with	Feed efficiency
Phylum	31213524	*Lentisphaerae*	Positively_correlated_with	Feed efficiency
Genus	33260665	*Methanobrevibacter*	Positively_correlated_with	Feed efficiency
Genus	31213524			Feed efficiency
Genus	31213524	*Mucispirillum*	Positively_correlated_with	Feed efficiency
Family	28526795	*Nocardiaceae (Rhodococcus)*	Negatively_correlated_with	Feed efficiency
Genus	36381065	*Oscillibacter*	Significantly_negatively_correlated	Feed efficiency
Genus	32698902	*Oscillibacter*	Significantly_correlated	Feed efficiency
Genus	28526795		Positively_correlated_with	Feed efficiency
Species	36381065	*Paraprevotella clara*	Significantly_positively_correlated	Feed efficiency
Family	30417245	*Peptococcaceae*	Negatively_correlated_with	Feed efficiency
Genus	34438645	*Peptococcus*	Significantly_correlated	Feed efficiency
Genus	32038603	*Prevotella*	Correlated_with	Feed efficiency
Genus	33839961		Positively_correlated_with	Feed efficiency
Species	34258424	*Prevotella* 9	Positively_correlated_with	Feed efficiency
Species	36381065	*Prevotella copri*	Significantly_positively_correlated	Feed efficiency
Family	33634901	*Prevotellaceae*	Significantly_correlated	Feed efficiency
Family	13			Feed efficiency
Species	34258424	*Prevotellaceae* TCG-001	Positively_correlated_with	Feed efficiency
Family	33634901	*Rikenellaceae*	Significantly_correlated	Feed efficiency
Genus	32295250	*Rothia*	Positively_correlated_with	Feed efficiency
Genus	29746643	*Ruminobacter*	Negatively_correlated_with	Feed efficiency
Family	33634901	*Ruminococcaceae*	Significantly_correlated	Feed efficiency
Family	32616095		Correlated_with	Feed efficiency
Family	31213524		Positively_correlated_with	Feed efficiency
Genus	32698902	*Ruminococcus*	Significantly_correlated	Feed efficiency
Genus	13			Feed efficiency
Species	32605690	*Ruminococcus flavefaciens*	Positively_correlated_with	Feed efficiency
Genus	29746643	*Shigella*	Negatively_correlated_with	Feed efficiency
Family	33634901	*Streptococcaceae*	Significantly_correlated	Feed efficiency
Family	32616095		Correlated_with	Feed efficiency
Genus	32295250	*Subdoligranulu*	Positively_correlated_with	Feed efficiency
Genus	33260665	*Treponema*	Positively_correlated_with	Feed efficiency
Species	32038603	*Treponema bryantii*	Correlated_with	Feed efficiency
Species	32038603	*Treponema porcinum*	Correlated_with	Feed efficiency
Genus	31291967	*Treponema_2*	Negatively_correlated_with	Feed efficiency
Genus	29746643	*Veillonella*	Negatively_correlated_with	Feed efficiency
Family	32616095	*Veillonellaceae*	Correlated_with	Feed efficiency

In the table, “significantly_correlated”, “significantly_positively_correlated”, “significantly_ negatively_correlated” are all labeled with *P* < 0.05 in the data source, and “upregulate” indicates a significant increase in the relationship, which is also determined based on *P* < 0.05 in the data source.

### Metabolic pathways involving gut microbiota

**Figure 3:**
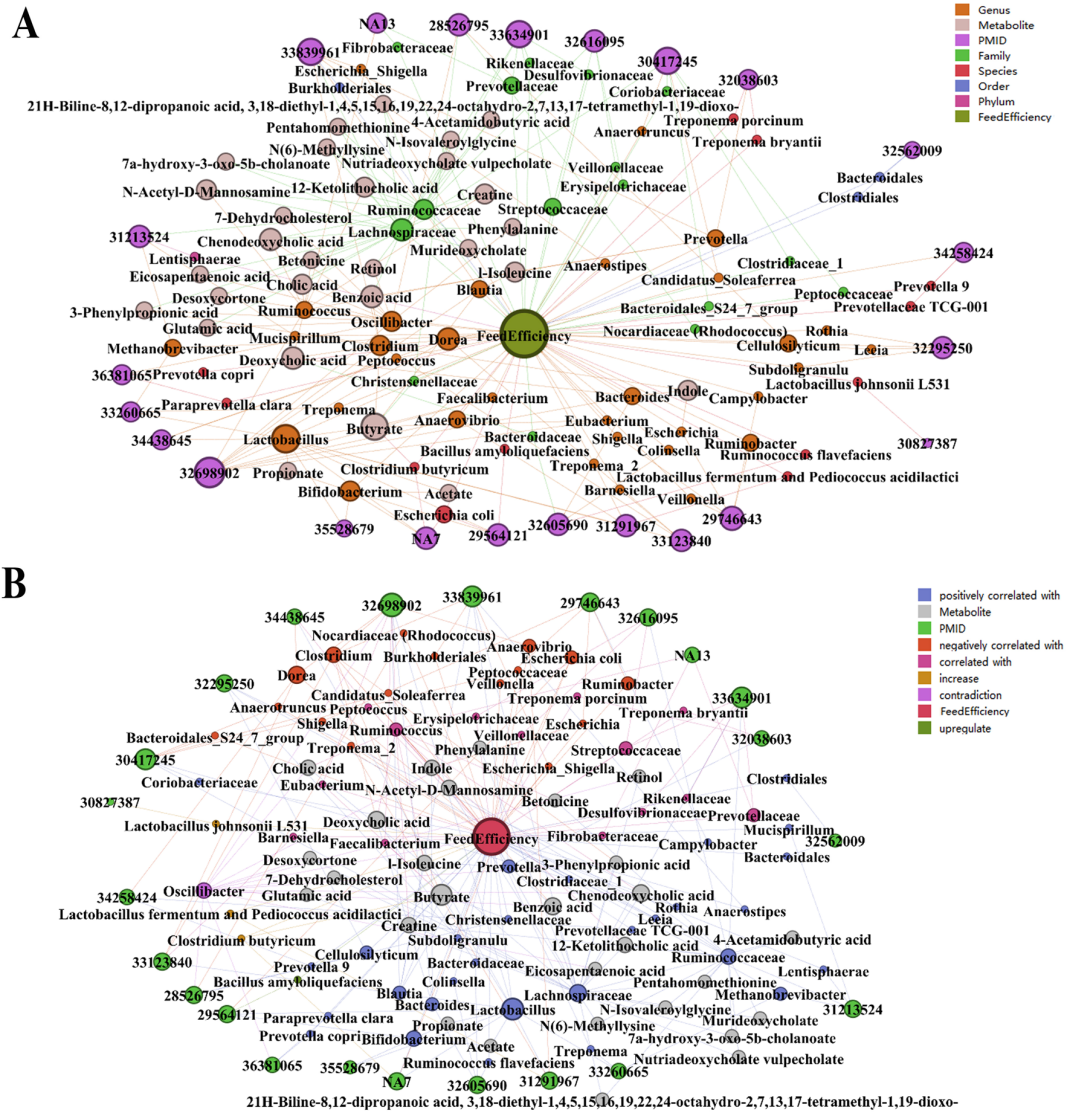
All metabolites associated with microbiota related to feed efficiency. In (**A**), each node represents an entity, in order to show the results more clearly, the different node colours represent the phylum, order, genus and species of microorganisms. In (**B**), as shown in the legend at the upper right corner, the different node colors represent the relationship between microorganisms and FE, positive correlation, negative correlation, correlation, increase, upregulate, etc. Then, the node size represents the number of connecting line, in other words, the larger the node, if there is more literature studying the microbe. In addition, only the nodes with two or more metabolites produced by microorganisms are shown in the figure.

**Figure 4:**
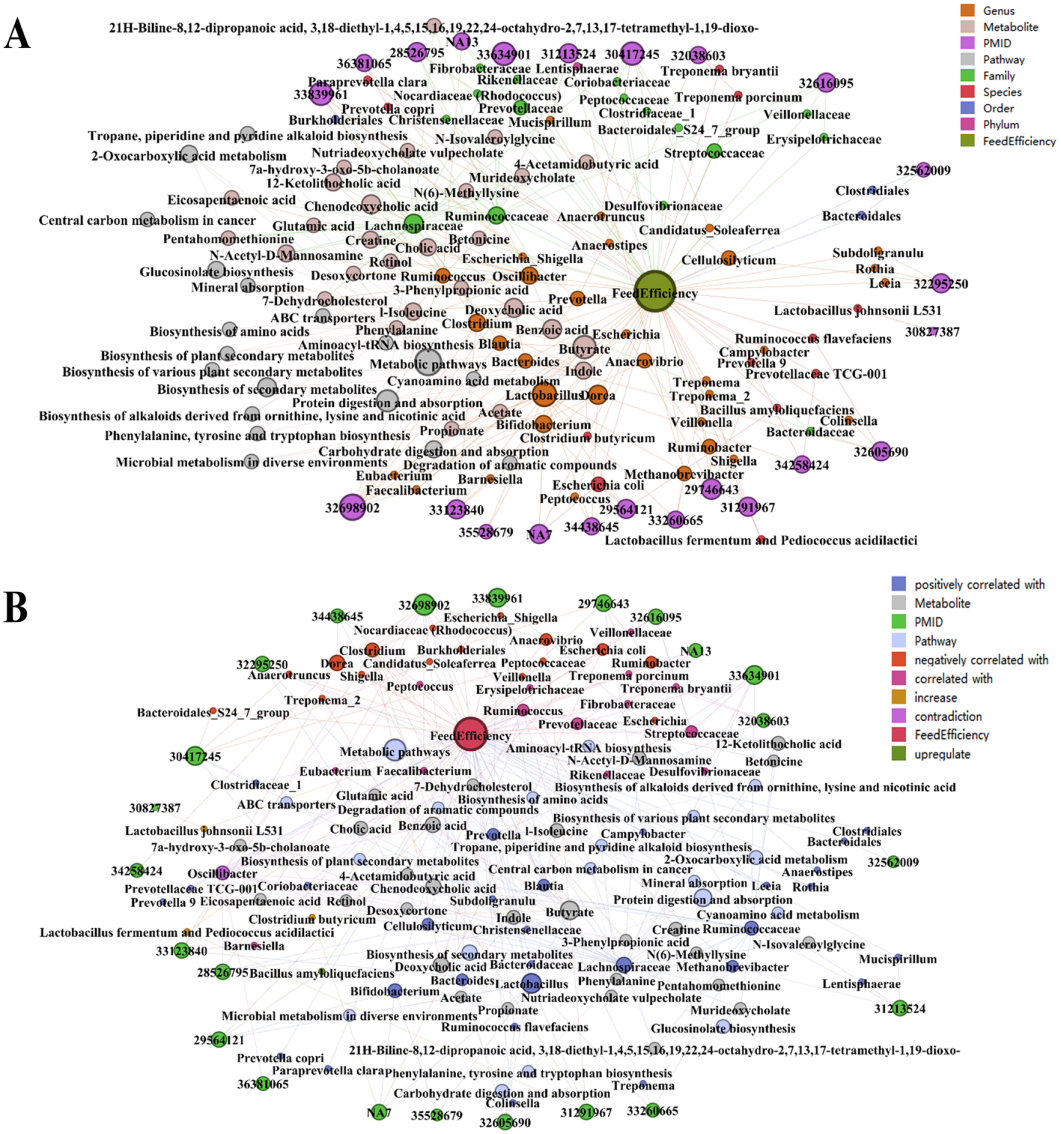
All metabolic pathways associated with microbiota related to feed efficiency. In (**A**), each node represents an entity, in order to show the results more clearly, the different node colours represent the phylum, order, genus and species of microorganisms. In (**B**), as shown in the legend at the upper right corner, the different node colors represent the relationship between microorganisms and FE, positive correlation, negative correlation, correlation, increase, upregulate, etc. Then, the node size represents the number of connecting line, in other words, the larger the node, if there is more literature studying the microbe. Otherwise, only metabolic pathways involving two or more metabolites were shown in the figure.

**Figure 5:**
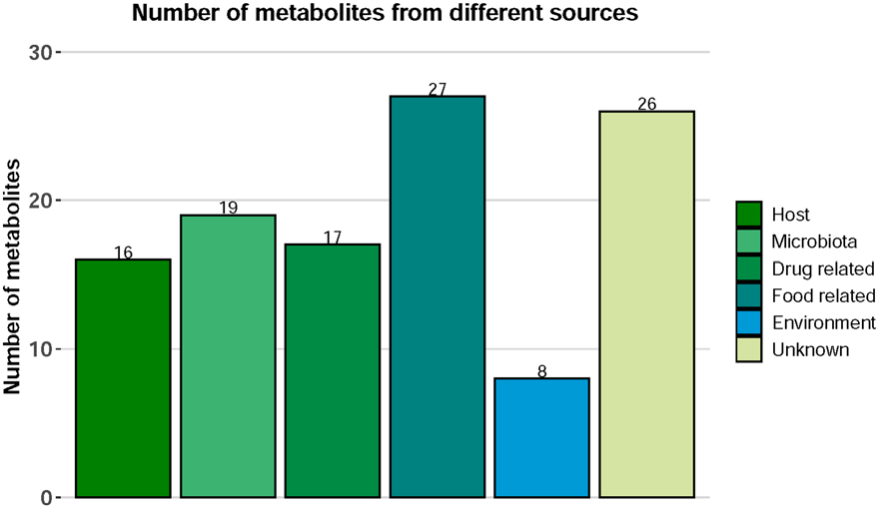
Number of metabolites from different sources.

**Figure 6:**
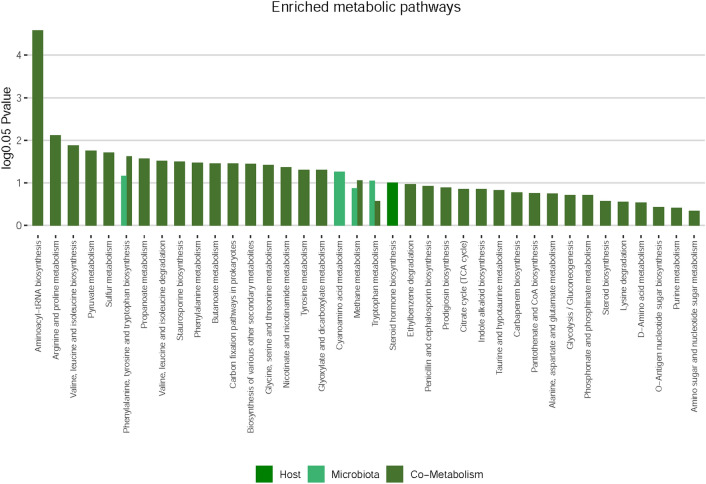
Functional enrichment analysis of metabolites.

Query results based on *pgmReading* and *pgmDatabase* show that 53 metabolites produced by these microorganisms mentioned in Fig. 2, including Butyrate (produced by *Blautia*, *Christensenellaceae*, *Clostridium*, *Clostridium butyricum*, *Eubacterium*, *Faecalibacterium* and *Lactobacillus*), Benzoic acid (produced by *Clostridium*, *Lachnospiraceae*, *Oscillibacter* and *Prevotella*), Chenodeoxycholic acid (produced by *Lachnospiraceae*, *Oscillibacter*, *Ruminococcaceae* and *Ruminococcus*), Deoxycholic acid, 12-Ketolithocholic acid, Cholic acid, Creatine, and other metabolites (Fig. 3 and Table S2). These metabolites are involved in 87 metabolic pathways such as Protein digestion and absorption, Biosynthesis of secondary metabolites, 2-Oxocarboxylic acid metabolism, etc. (Fig. 4). In addition, MetOrigin^25^ was performed to further analyze the origins of the metabolite and their functional enrichment. The diagram illustrates that 16 of the metabolites produced related to FE are from the host, 19 are from microbes, and 15 are common to both. It is worth noting that 27 of these metabolites are related to feed, accounting for 50.94% of the total (Fig. 5). Functional enrichment analysis of metabolites showed that they were mainly involved in metabolic pathways such as Aminoacyl-tRNA biosynthesis, Arginine and proline metabolism, Valine, leucine and isoleucine biosynthesis (Fig. 6). Among them, the metabolic pathway in which the host participates alone is Steroid hormone biosynthesis, and the metabolic pathway in which the microbes participate alone is Cyanoamino acid metabolism. Most of the pathways are co-metabolism between the host and the microbes.

### Factors significantly related to feed efficiency in PGMKG

**Figure 7:**
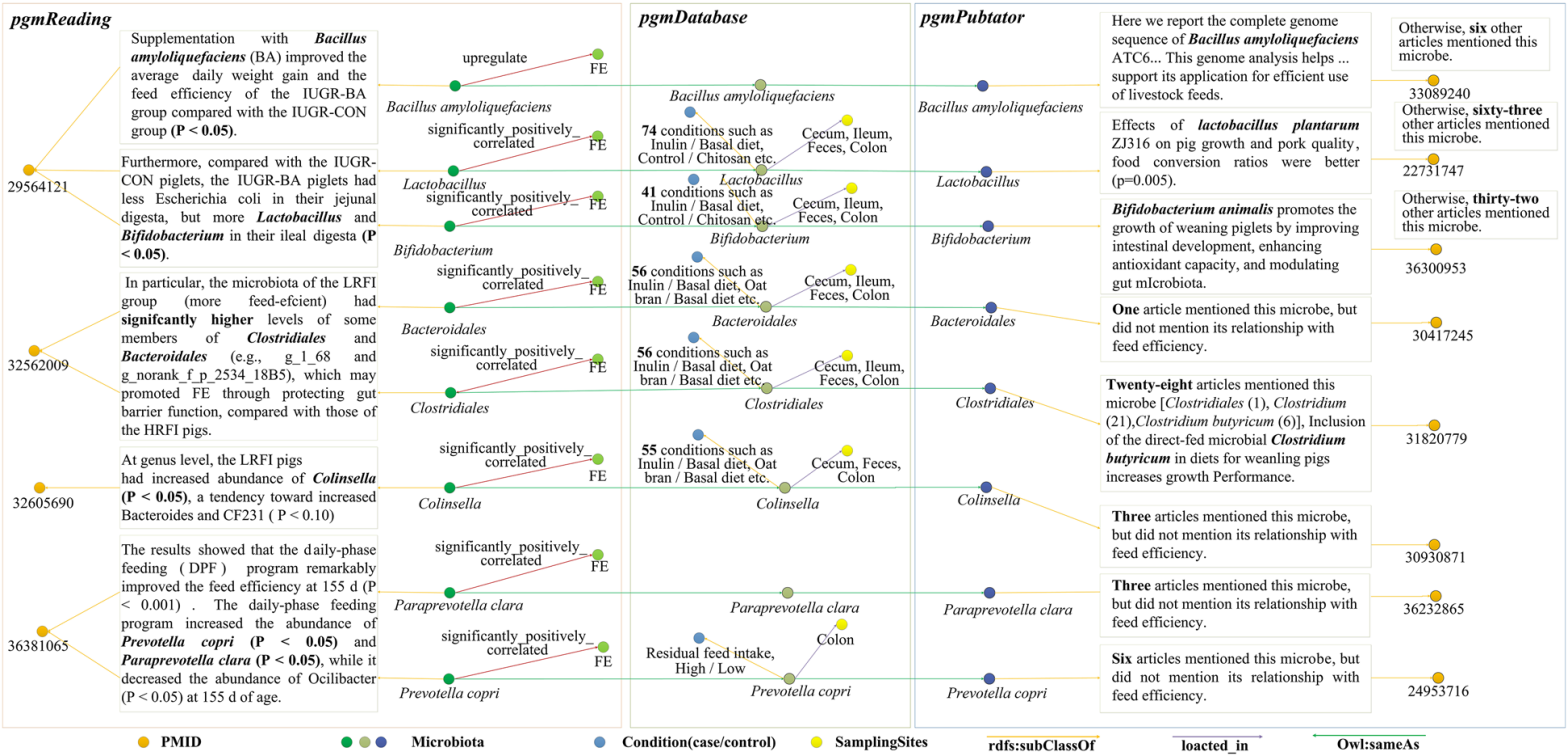
All factors significantly positively associated with microbiota related to feed efficiency in PGMKG.

In this section, we design query case 3 to gain knowledge on microbes that are significantly related to feed efficiency. In order to fully understand the current state of research on microorganisms significantly associated with feed efficiency, as well as to demonstrate the complementary relationship of our three knowledge bases, we have plotted Fig. 7. In the *pgmReading* knowledge base, it can be concluded that the addition of *Bacillus amyloliquefaciens* to the feed significantly improved the feed efficiency of the experimental group compared to the control group, *Lactobacillus* and *Bifidobacterium* appeared more frequently (*P* < 0.05) in the ieal digesta of the experimental group, and *pgmDatabase* yielded that *Lactobacillus* was associated with feed efficiency under 74 conditions (case/control) such as Inulin/Basal diet, Conrrol/Chitosan etc. were all associated with feed efficiency. In the *pgmPubtator*, *Lactobacillus plantarum* can be found to play a role in improving feed conversion efficiency, with 63 remaining articles mentioning *Lactobacillus*. Similarly, the microbes *Bifidobacterium*, *Bacteroidales*, *Clostridiales*, *Colinsella*, *Paraprevotella clara* and *Prevotella copri* were significantly positively correlated with feed efficiency, which we derived from *pgmReading* knowledge base. These microbes were almost all found in the *pgmDatabase*, *Bifidobacterium* (41 conditions—case/control), *Bacteroidales* (56 conditions—case/control), *Clostridiales* (56 conditions—case/control), *Colinsella* (55 conditions—case/control), and *Prevotella copri* (Residual feed intake, High/Low) were related to FE. Besides, *Bifidobacterium* (32 articles), *Bacteroidales* (1 articles), *Clostridiales* (1 articles), *Colinsella* (3 articles), *Paraprevotella clara* (3 articles) and *Prevotella copri* (6 articles) were mentioned in the *pgmPubtator* knowledge base (Table S3), additionally, it was found that experiments have been conducted to validate the role of *Bifidobacterium animalis, Clostridium butyricum* in promoting the growth of weaned piglets.

### Microbes associated with specific types of feed or pig breeds

To demonstrate that our graph not only supports simple queries but also enables complex queries, we have designed two complex query cases. Our annotation logic is based on the idea that under certain experimental conditions, pigs exhibit changes in traits such as growth, reproduction, and disease, as well as changes in gut microbiota. This is reflected in the graph with a core path as follows: (FeedadditivesName, belong_to, ExperimentDesign)—(ExperimentDesign, belong_to, SwineBreed)—(ExperimentGroup, belong_to, ExperimentDesign)—(ExperimentGroup, change, MicrobiotaName)—(ExperimentGroup, change, GrowthPerformance), etc.

From this, we can find that:

Query Case 4: Given the feed additive type, specifically FeedadditivesName as "fermented spent mushroom substrates," we first determine which experimental design it belongs to, then obtain the specific experimental groups and the microbiota affected by these groups. The query reveals that the affected microbes include *Clostridium disporicum*, *Lactobacillus gasseri*, *Roseburia*, *Streptococcus*, *Lactobacillus*, *Bacteroidetes*, and *Firmicutes*.

Query Case 5: Given the pig breed, we first determine what experimental designs have been applied to this breed. Based on this, we then identify the related microbiota. The results show that the associated microbes are *Bacteroidetes* and *Firmicutes*.

**Table:** 

Query case 4. The Cypher query code for the microbes associated with fermented feed additives like fermented spent mushroom substrates MATCH (n1:FeedFermentationType{name:'fermented spent mushroom substrates'})-[r1] → (m1:ExperimentDesign) return n1, r1, m1 r1: belong_to, m1: fed basal diets supplemented with 3% FSMS MATCH (n2:ExperimentGroup)-[r1] → (m1:ExperimentDesign{name:'fed basal diets supplemented with 3% FSMS'}) return n2, r1, m1 r1: belong_to, n2: FSMS MATCH (n2:ExperimentGroup{name:'FSMS'})-[r2] → (m3:MicrobiotaName) return n2,r2,m3 r2: influence, m3: *Clostridium disporicum*, *Lactobacillus gasseri*, *Roseburia, Streptococcus*, *Lactobacillus*, *Bacteroidetes*, *Firmicutes*	

**Table:** 

Query case 5. The Cypher query code for the microbes associated with Duroc × Large White × Landrace MATCH (n1:ExperimentDesign)-[r1] → (m1:SwineBreed{name:'Duroc × Large White × Landrace'}) return n1, r1, m1 r1: belong_to, n1: control plus oregano and tributyrin, control plus methyl salicylate and tributyrin, control plus antibiotics, basal diet MATCH (m2:ExperimentGroup)-[r1] → (n1:ExperimentDesign{name:'control plus oregano and tributyrin'}) return m2, r1, n1 r1: belong_to, m2: OT MATCH (m2:ExperimentGroup{name:'OT'})-[r2] → (n3:MicrobiotaName) return m2, r2, n3 n3: *Bacaeroides*, *Firmicutes*	

### Evaluation of the capability of PGMKG

To provide a comprehensive evaluation, we have collected 30 relevant questions and answers from researchers in this field (Table S4). We then queried our knowledge graph based on each question to verify the query efficiency of the graph. Manual calculation of metrics such as recall, precision, and F1 score:

Recall measures how many correct answers are returned by PIMKG out of the expected results. The formula for recall is: Recall = Number of correct answers/Number of expected results = 25/30 = 83.3%.

Precision measures how many correct answers are returned by PIMKG out of the total number of returned results. The formula for precision is: Precision = Number of correct answers/Number of returned results by PIMKG = 25/28 = 89.3%.

F1 score combines both recall and precision into a single evaluation metric. The formula for F1 score is: F1 score = 2 × (Precision × Recall)/(Precision + Recall) = 0.94.

## Discussion

The utilization of knowledge graph in the field of pig gut microbiota and pig feeding are still in its early stages, but holds great potential for the design of animal feed formulas targeting gut microbiota, and pig husbandry management. Our knowledge graph, PGMKG, systematically maps the relationships between pig gut microbes and feed efficiency and the associated metabolites and metabolic pathways, this will provide important insights and implications for both research and practical applications in pig production. Specifically designed to support the optimization of feed efficiency in pigs, our knowledge graph highlights key microbial species and their metabolic pathways that influence feed efficiency. It offers a comprehensive and integrated view of these factors, facilitating the identification of potential targets for interventions and enabling the formulation of more effective strategies to improve feed efficiency. For instance, based on our knowledge graph, we have identified 25 microorganisms positively associated with feed efficiency. These associations were extracted from various studies: Zhang et al.^26^ and PMID: 35528679 (*Bifidobacterium*, *Lactobacillus*), PMID: 28526795 (*Cellulosilyticum, Christensenellaceae, Oscillibacter*), PMID: 29746643 (*Campylobacter*), PMID: 30417245 (*Clostridiaceae_1, Coriobacteriaceae, Lachnospiraceae*), PMID: 31213524 (*Lentisphaerae, Methanobrevibacter, Mucispirillum, Ruminococcaceae*), PMID: 31291967 (*Lactobacillus*), PMID: 32295250 (*Cellulosilyticum, Leeia, Rothia, Subdoligranulu*), PMID: 32605690 (*Ruminococcus flavefaciens, Bacteroidaceae, Bacteroides*), PMID: 33260665 (*Lactobacillus, Methanobrevibacter, Treponema*), PMID: 33839961 (*Anaerostipes, Blautia, Lachnospiraceae, Prevotella*), and PMID: 34258424 (*Prevotella 9, Prevotellaceae TCG-001*). Among these studies, for example, when 0.1% *Clostridium butyricum* was added to the feed of weaned piglets, the gain-to-feed ratio increased, and the quantities of *Bifidobacterium* and *Lactobacillus* in feces also increased^26^. Enzymatic hydrolysis of tuna dark muscle improved feed efficiency and increased the relative abundance of *Bifidobacterium* and *Lactobacillus*^27^. The combined addition of *Lactobacillus fermentum* and *Pediococcus acidilactici* to feed improved the feed-to-gain ratio (F/G) and promoted the presence of *Lactobacillus* in the caecal digesta^28^. Adding 2% glycine to the feed increased the feed conversion ratio and the abundance of *Anaerostipes, Blautia, Lachnospiraceae*, and *Prevotella* in the colon^29^. Dietary chenodeoxycholic acid improved feed efficiency and increased the relative abundance of *Prevotella 9* and *Prevotellaceae TCG-001*^30^. Therefore, it can be inferred that *Bifidobacterium*, *Lactobacillus*, *Anaerostipes*, *Blautia*, *Lachnospiraceae*, *Prevotella*, *Prevotella 9*, and *Prevotellaceae TCG-001* are positively associated with feed efficiency. These relationships are inferred from side observations and may require further experimental validation. Moreover, studies have shown that in high feed efficiency pigs, the fecal content of *Cellulosilyticum*^31^, *Christensenellaceae*^31^, *Oscillibacter*^31^, *Campylobacter*^32^, caecal content of *Clostridiaceae_1*^33^, *Coriobacteriaceae*^33^, *Lachnospiraceae*^33^, and fecal or caecal content of *Lentisphaerae*^8^, *Methanobrevibacter*^8^, *Mucispirillum*^8^, *Ruminococcaceae*^8^, *Cellulosilyticum*^34^, *Leeia*^34^, *Rothia*^34^, *Subdoligranulu*^34^, *Ruminococcus flavefaciens*^35^, *Bacteroidaceae*^35^, *Collinsella*^35^, *Bacteroides*^35^, *Lactobacillus* ^2^in the large intestine, *Methanobrevibacter* ^2^in the small and large intestines, and *Treponema*^2^ is relatively abundant. This directly indicates a positive correlation between these microorganisms and feed efficiency.

Similarly, there are 16 microorganisms negatively associated with feed efficiency. These associations were extracted from various studies: Zhang et al.^26^ (Escherichia coli), PMID: 28526795 (*Nocardiaceae (Rhodococcus)*), PMID: 29746643 (*Escherichia/Shigella, Ruminobacter, Veillonella*), PMID: 30417245 (*Anaerotruncus, Bacteroidales_S24_7_group, Candidatus_Soleaferrea, Peptococcaceae*), PMID: 31291967 (*Anaerovibrio, Treponema_2*), PMID: 32038603 (*Prevotella*), PMID: 33839961 (*Burkholderiales, Clostridium, Escherichia–Shigella*), and PMID: 34258424 (*Dorea*). Among these studies, for example, when 0.1% *Clostridium butyricum* was added to the feed of weaned piglets, the gain-to-feed ratio increased, and the quantity of Escherichia coli in feces decreased^26^. The combined addition of *Lactobacillus fermentum* and *Pediococcus acidilactici* to feed improved the feed-to-gain ratio (F/G) and inhibited *Anaerovibrio* and *Treponema_2* in the caecal digesta^28^. Adding 2% glycine to the feed increased the feed conversion ratio and decreased the abundance of *Burkholderiales*, *Clostridium*, and *Escherichia–Shigella* in the colon^29^. Dietary chenodeoxycholic acid improved feed efficiency and reduced the relative abundance of *Dorea*^30^.Therefore, it can be inferred that *Escherichia coli*, *Anaerovibrio*, *Treponema_2*, *Burkholderiales*, *Clostridium*, and *Escherichia–Shigella* are negatively associated with feed efficiency. These relationships may require further experimental validation. Moreover, studies have shown that in low feed efficiency pigs, the abundance of *Nocardiaceae (Rhodococcus)*^31^, *Escherichia/Shigella*^32^, *Ruminobacter*^32^, *Veillonella*^32^, *Anaerotruncus*^33^, *Bacteroidales_S24_7_group*^33^, *Candidatus_Soleaferrea*^33^, *Peptococcaceae*^33^, and *Prevotella* in the caecum^36^ is relatively high. This directly indicates a negative correlation between these microorganisms and feed efficiency.

Most of the microorganisms positively associated with feed efficiency are probiotics, such as *Bifidobacterium* and *Lactobacillus*. Probiotics play a crucial role in regulating gut microbiota, host immune responses, and nutrient digestibility, reducing diarrhea, and providing antitoxin effects, thereby improving the overall health of pigs^37^. The role of bacteria in nutrient processing and energy harvesting in the host is also significant. Many microorganisms, like *Christensenellaceae*, *Treponema* and *Methanobrevibacter* are involved in the degradation of carbohydrates and the breakdown of plant-derived polysaccharides, producing short-chain fatty acids (SCFAs) that supply energy to pigs. Additionally, *Treponema* and *Methanobrevibacter* are related to fiber digestibility, breaking down indigestible substances into usable energy^2^. Butyrate, a metabolic product, increases energy expenditure and reduces food intake^38^, closely linked to high feed efficiency. Therefore, butyrate-producing microorganisms such as *Ruminococcus* and *Lachnospiraceae* are enriched in pigs with higher feed efficiency^2^. Various metabolic pathways regulated by the gut microbiota are crucial for pig feed efficiency. Besides providing high-quality protein in feed, these microorganisms are vital for the absorption and transport of amino acids, ensuring their effective use for protein synthesis and growth, especially essential amino acids like lysine, threonine, tryptophan, and arginine^38^. To ensure effective glucose utilization and prevent excessive fat storage, the host must regulate glucose metabolism^38^. Microorganisms such as *Bacteroides* and *Lactobacillus* are involved in metabolic pathways like carbohydrate digestion and absorption, glycolysis/gluconeogenesis, and the glyoxylate and dicarboxylate metabolism. Under optimal conditions, these microorganisms improve the gut environment, enhance the gut barrier, promote digestion and absorption, increase feed efficiency, and boost the pig’s immunity. Conversely, microorganisms negatively associated with feed efficiency, like *Escherichia coli*, *Prevotella*, and *Escherichia–Shigella*, often carry pathogenic properties or compete with the host for nutrients^2^. Researchers can utilize this information to set appropriate experimental conditions tailored to their research objectives, thereby achieving more precise and effective outcomes.

This study refined the pig gut microbiota domain ontology, based on the previous ontology of swine gut microbiota used for federal queries^39^, we have developed a more comprehensive and versatile framework, which defined a wider range of concepts and more comprehensive applications. The ontology is mainly used to solve the interoperability between heterogeneous data from multiple sources^40^, and has also been increasingly employed in agricultural field. For example, similar ontology model for describing aquaponics systems was constructed by Abbasi et al. to support aquaponics farm production facility layout and system design^15^. A potato ontology was constructed for potato production environments for automated decision support systems and data exchange tasks in the potato industry^41^. We made a canonical, standardized ontology for the field of pig gut microbiology to develop a literature-driven knowledge graph of pig gut microbiota.

Furthermore, the PGMKG has enabled the identification of specific metabolic pathways and metabolites produced by these key microbes. For example, the production of butyrate by various microbes like *Blautia* and *Clostridium butyricum* highlights a potential mechanism through which gut microbiota can influence FE. These insights into metabolic pathways can help in developing more targeted and efficient feed additives or probiotics that can modulate the gut microbiota for optimal FE. Methodologically, we obtained 2307 entities and 6217 triples by careful reading and manual labelling of 157 documents, created the *pgmReading* knowledge base, and verified the data sources several times to ensure the accuracy and authenticity of the knowledge graph. In addition, the PubTator tool was utilized to automatically identify microbial concepts^42^ and create a *pgmPubtator* knowledge base to enrich microbial data. By combining the ADDAGMA^43^, gutMGene^44^ and KEGG^45^ databases, we established the *pgmDatabase* knowledge base, providing a comprehensive understanding of the interplay between gut microbes and hosts, including the metabolic pathways they regulate. Ultimately, we combined all three to construct the knowledge graph of pig gut microbiota-PGMKG, a dynamic and scalable tool that not only emphasizes the effect of gut microbiota on feed efficiency but also seamlessly integrates the latest research. Overall, our database integrates various types of data, including experimental design, feed composition, environmental factors, and growth traits. It also incorporates unique relationships such as environmental impacts and host–microbiota interactions, which are not commonly found in existing knowledge bases. Moreover, our knowledge graph uses Neo4j as the back-end of storing data resources, and GraphXR as the front-end of visual display and query, which provides users with a pleasant experience. Using the basic Cypher query language, we can obtain results and export them in csv, excel, gif, png formats. Crucially, our PGMKG stands out by capturing both explicit and implicit relationships. While explicit connections are directly extracted using the Cypher query language, the implicit ones are innovatively deduced through the fusion of three knowledge bases, highlighting the depth and breadth of our research's innovation. Additionally, based on the question–answer pairs provided by researchers in this field, our knowledge graph demonstrates a strong capability to answer various queries. Apart from being unable to respond to non-existent associations and undefined indicators, the graph can provide answers to routine questions such as common feed additives, evaluation indicators of feed nutritional value, typical sampling locations in the intestine, metabolic pathways regulating feed efficiency, and more. Compared to traditional databases, our knowledge graph offers enhanced readability and intuitive visualization of complex relationships. It provides an interactive platform that allows researchers to explore data dynamically, uncovering insights that might be missed in static databases.

Due to the diversity of microbes and the speed of updating of the literature, we were not able to fully cover the entire field and the number of entities and relationships we extracted was limited. The potential of our graph is not yet complete as we have not adopted machine learning algorithms for in-depth mining and reasoning. We plan to enhance our research framework by improving data coverage and accuracy, refining semantic reasoning algorithms, and integrating real-time data updates. These improvements will broaden the utility of our research, making it more robust reference for the subsequent mining and deep learning of functional microbes. For instance, by identifying microbial species associated with specific feed types and pig breeds, PGMKG can inform the development of customized feed formulations to optimize pig health and production performance; PGMKG can aid in the identification of microbial markers associated with disease susceptibility and resilience, facilitating early detection and targeted interventions for disease prevention and control; By uncovering associations between microbial composition and desirable phenotypic traits, PGMKG can support the selection of breeding stock with improved health, productivity, and feed efficiency. Additionally, incorporating more real-world production information into the knowledge graph can lead to the development of agricultural big models in the field of swine health farming or disease prevention and control, effectively addressing questions from researchers and laborers, thereby meeting practical needs.

## Conclusion

The PGMKG represents a significant advancement in our understanding of the relationship between pig gut microbiota and FE. Our preliminary graph shows that *Bacillus amyloliquefaciens*, *Clostridium butyricum, Lactobacillus fermentum and Pediococcus acidilactici*, *Lactobacillus johnsonii L531* can increase FE under certain conditions. This insight lays a foundation for further exploration of functional gut microbes and provides a basis for experimental validation. Most importantly, PGMKG can also be used as an example for future research on major performance traits such as early-weaning stress alleviation and fat deposition in pigs. In addition, we can lay the foundation for predicting the relationship between gut microbes and traits in pigs.

## Methods

### Manual curation of the *pgmReading* knowledge base

To construct the* pgmReading* knowledge base, we searched the Web of Science (WOS) from January 1, 2000 to October 31, 2022 based on the following search formula: TS = (“pig” OR “swine” OR “piglet”) AND TS = (“feed efficiency” OR “feed conversion efficiency” OR “feed conversion ratio”) AND TS = (“gut microbiota” OR “intestinal microorganisms” OR “intestinal microbiota” OR “intestinal microbes”), a total of 280 articles were downloaded, and 157 articles were screened by manual reading.

First, we carefully read 157 articles describing the relationship between pig intestinal microbiota and feed efficiency and manually listed the entities, attributes, and relationships, and labeled their abstracts using the label studio platform. For example, we extracted entity types from the sentence " A total of 180 healthy piglets (Duroc × [Landrace × Yorkshire]; weighing 7.81 ± 1.51 kg each, weaned at d 28) were randomly divided into 5 treatments "^46^. The entity types extracted from this sentence include: Swine_Breed: Duroc × [Landrace × Yorkshire]; Swine_Stage: piglets; Swine_Age: weaned at d 28; Swine_Weight: 7.81 ± 1.51 kg. All the annotations were performed in the format of the Swine Gut Microbiota domain ontology format, as shown in Fig. 1. The entity types extracted from these 157 papers were Swine, Microbiota, Feed Efficiency, Index, Gene, Protein, Metabolism, Experiment, Feed, Disease and Environment (Table 3). Next, weexported the annotated results in JSON format and then process them into the format "Triple_list": {"relation": "", "object_type": "", "subject_type": "", "object": "", "subject": ""}. This was imported into Neo4j as the *pgmPubtator* knowledge base.

**Table 3:** Entities, attributes and relations in *pgmReading* knowledge base.

Class	Element	Detail type
Entity type	Swine	SwineBreed/SwineSex/SwineWeight/SwineAge/SwineStage/SwinePlace/SwineType
	Microbiota	MicrobiotaName/MicrobiotaTaxonomy/MicrobiotaDiversity/MicrobiotaType
	FeedEfficiency	FeedEfficiency/ResidualFeedIntake/FeedConversionRatio
	Performance	CarcassTraits/GrowthPerformance/SerumIndex/AcidType/DigestibilityType/UtilizationefficiencyType/ConcentrationType/AminoacidType/AminoacidName/CellType/CellName
	Gene	GeneType/GeneName
	Protein	ProteinType/ProteinName
	Metabolism	MetabolismType/MetabolismName/MetabolitesType/MetabolitesName
	Experiment	ExperimentDesign/ExperimentGroup/ExperimentDays/SamplingSites/SamplingTissues/SamplingType
	Feed	DietType/NutrientType/FeedsubstituteType/FeedsubstituteName/SubstituteAmount/FeedingredientName/FeedingredientType/FeedFermentationType/Antibiotic/StarchType/FeedadditivesType/FeedadditivesName/AntibioticSubstituteName/AntibioticSubstituteType
	Disease	DiseaseName/DiseaseType
	Environment	VaccineType/WaterTemperature/ConditionTemperature/ContaminantType/ContaminantName
Relation type	Owl:sameAs	equal_to/similar
	rdf:hasProperty	has_Time/has_Breed/has_Stage/has_Sex/has_Age/has_Day/has_Weight/has_Amonut/has_Place/has_Food
	rdfs:subClassOf	belong_to
	Regulation	increase/decrease/upregulate/downregulate
	Treatment effect	significantly_positively_correlated/significantly_negatively_correlated/significantly_correlated/correlated_with/positively_correlated_with/negatively_correlated_with/modulate/involved_in/is_carried_out_by/express/influence/noinfluence/located_in/vaccinated_with/inhibite/dominated/followed/feed/produce
	Comparison	higher/heavier/highest/intermediate/significantly_higher/lower/lowest/high_diversity/significant_diferences/nosignifcant_diferences

### Automatic generation of the *pgmPubtator* knowledge base

To make the data more complete, we retrieved 65,412 articles from PubMed using the keyword gut microbiota, and used PubTator^42^ to automatically identify and extract the entities in the titles and abstracts of the articles, which can identify the Gene, Disease, Chemical, Mutation, Specie in the literature, etc., as well as the Taxonomy ID, Medical Subject Headings (MeSH) ID, and other ID of these entities, based on the need of *pgmPubtator* knowledge base construction, we only keep the microbes and their IDs to be stored in the form of triples. The *pgmPubtator* knowledge base is constructed through the following steps: firstly, we search for "gut microbiota" on the PubTator tool page to obtain a .pubtator file. This file contains various details such as PMID (article identifiers), entity positions in sentences, entity types, Taxonomy ID, MeSH, and more. Next, we save this file in CSV format and extract the relevant microbiota information. Then, we store this information in the form of triples (MicrobiotaName, hasSource, PMID) and (MicrobiotaName, hasID, PMID). Finally, we import this extracted data into the pre-existing *pgmReading* graph, integrating it with the existing data.

### Semi-automatic construction of the *pgmDatabase* knowledge base

In order to expand the information related to microbes, we collected information on microbes related to feed efficiency in ADDAGMA^43^, and looked up the metabolites of microbes in gutMGene^44^, and investigated the metabolic pathways that these metabolites are involved in KEGG^45^, and other information. These databases were assembled into the *pgmDatabase* knowledge base through ontology mapping. The specific process of building the *pgmDatabase* knowledge base is as follows: first of all, we download data from the ADDAGMA official website, which provides microbiota phenotype association data for four animal species: pigs, cows, horses, and chickens, from a collection of 356 publications. Since our focus is on the correlation between pig gut microbiota and feed efficiency, we performed an initial screening of the data. Then, we imported the filtered data into the knowledge graph in the form of triples such as (MicrobiotaName, belong_to MicrobiotaTaxonomy), (MicrobiotaName, correlated_with, FE), and (MicrobiotaName, located_in, SamplingSites). Additionally, other information from the table, such as Condition (case/control), Pvalue, Mean(RA)_control, is stored as attributes of MicrobiotaName. Subsequently, after obtaining the gutMGene information, we imported it into Neo4j in the form of triples (MicrobiotaName, produce, MetabolitesName). Similarly, information from KEGG was integrated into the knowledge base in the form of triples (MetabolitesName, involved_in, MetabolismName). In the end, ontology mapping was performed to name the data according to the concepts defined in the ontology. This ensures data standardization, compatibility, and eliminates redundancy.

### Knowledge base integration and Cypher queries

We integrate *pgmReading*, *pgmPubtator* and *pgmDatabase* knowledge bases into Neo4j, which can integrate three independent knowledge bases and perform search and reasoning through the Cypher query language. Query case 1 queries the *pgmReading* knowledge base for all microbes related to feed efficiency. Query case 2 queries the *pgmDatabase* knowledge base for all metabolites and metabolic pathway. Query case 3 queries all factors significantly positively associated with microbiota related to feed efficiency in PGMKG. Query case 4 queries the microbes associated with fermented feed additives like fermented spent mushroom substrates. Query case 5 queries the microbes associated with Duroc × Large White × Landrace. The **match** is used to search for nodes and relationships that satisfy a certain condition, and **return** can return the results of the query to the user, **where** and **match** are used together to act as a filter. In addition, GraphXR was used to connect Neo4j for displaying and querying knowledge graph of pig gut microbiota, and Gephi was used to visualize the query results.

### Verification of accuracy and effectiveness of PGMKG

Firstly, we used logical reasoning to detect logical errors and inconsistencies within the graph, and employed ontology constraints and type constraint checks to ensure the data conforms to predefined ontological specifications. Secondly, we invited domain experts to review the key nodes and relationships in the knowledge graph. The experts thoroughly examined important entities and relationships, provided feedback, and helped correct any potential errors. Then, to provide a comprehensive evaluation, we collected 30 relevant questions and answers from researchers in the field. Using our knowledge graph, we conducted queries in Cypher language embedded in Neo4j to verify the query efficiency and recall of the graph. Furthermore, the knowledge graph is subject to a regular update and maintenance schedule, continuously synchronizing with data sources to ensure the data's timeliness and accuracy. Additionally, we employ various metrics to assess the quality of the knowledge graph and have established a user feedback system, users can report errors, suggest new content, or propose improvements to existing content. Finally, we regularly review and incorporate user feedback to continually enhance the knowledge graph.

### Data privacy and copyright issues

Regarding data privacy, any personal or sensitive information obtained from sources such as PubTator, ADDAGMA or gutMGene is handled in accordance with applicable data protection laws and regulations. Personal identifiers are anonymized or removed to ensure the privacy and confidentiality of individuals involved in the research data. The authors ensure that all data used in PGMKG comply with ethical guidelines and regulations related to data privacy.

Regarding copyright issues, the authors respect the intellectual property rights of the original content creators and publishers. In cases where copyrighted material is used, appropriate permissions, licenses, or fair use provisions are obtained or followed. Proper citations are provided to attribute the original sources.

### Supplementary Information

Supplementary Legends.

Supplementary Table S1.

Supplementary Table S2.

Supplementary Table S3.

Supplementary Table S4.

## Data Availability

All data generated or analysed during this study are included in this published article and its supplementary information files.
