# Supplementary material for: Knowledge graph-derived feed efficiency analysis via pig gut microbiota
Source: Sci Rep. 2024 Jun 17;14:13939. doi: 10.1038/s41598-024-64835-6 (PMC11182767; doi:10.1038/s41598-024-64835-6)
Supplement: Supplementary file 1 — Supplementary Legends. [file 41598_2024_64835_MOESM1_ESM.docx]

**Supplementary Information**

Table S1. Sources of all microbiota associated with feed efficiency.

Table S2. All metabolites and pathway associated with microbiota related to feed efficiency.

Table S3. All factors associated with microbiota related to feed efficiency in PGMKG.

Table S4. A collection of thirty test questions.
