# Supplementary material for: Knowledge graph-derived feed efficiency analysis via pig gut microbiota
Source: Sci Rep. 2024 Jun 17;14:13939. doi: 10.1038/s41598-024-64835-6 (PMC11182767; doi:10.1038/s41598-024-64835-6)
Supplement: Supplementary file 5 — Supplementary Table S4. [file 41598_2024_64835_MOESM5_ESM.docx]

1. **Question:** What pig breeds are used to study the relationship between the pig gut microbiome and feed efficiency? **Answer:** Large White, Duroc, Landrace, etc.

2. **Question:** At what age stages of pigs have gut microbiome changes been studied? **Answer:** Piglets, growing-finishing pigs, adult pigs.

3. **Question:** What are the common types of feed additives? **Answer:** Trace elements, vitamins, amino acids, Chinese herbal medicine, probiotics, etc.

4. **Question:** What experimental grouping methods are mentioned in the literature? **Answer:** Control and experimental groupings, different feed component groupings.

5. **Question:** What fermented feeds are commonly used in experimental designs? **Answer:** Cassava residue, straw-processed feed, cottonseed meal, fruit residue, distiller's grains, rice bran, etc.

6. **Question:** What metabolic pathways might regulate feed efficiency? **Answer:** Carbohydrate metabolism, amino acid metabolism, glucose metabolism, glycolysis/gluconeogenesis, tricarboxylic acid cycle, cellulose degradation, etc.

7. **Question:** What metabolites are involved in the studies? **Answer:** Butyrate, short-chain fatty acids, benzoic acid, glyoxylate, dicarboxylate, etc.

8. **Question:** What diseases are related to changes in the gut microbiome? **Answer:** Enteritis, diarrhea.

9. **Question:** What carcass traits might be involved in the studies? **Answer:** Carcass weight, slaughter rate, carcass length, backfat thickness, loin eye area, lean meat percentage, fat percentage, etc.

10. **Question:** What are the evaluation indicators of pig gut health? **Answer:** Villus height, crypt depth, villus height to crypt depth ratio, intestinal cells, etc.

11. **Question:** What are the commonly used antibiotic alternatives? **Answer:** Probiotics, plant extracts, acidifiers, oligosaccharides, enzyme preparations, lysozyme, bacteriophages, etc.

12. **Question:** What genes might be mentioned in the studies? **Answer: *TNF-α, TGF-β, IFN-γ, IL-2, IL-1β, IL-6, IL-10, macB*, etc.**

13. **Question:** What microorganisms frequently appear in the studies? **Answer:** *Lactobacillus*, *Bifidobacterium*, *E. coli*, *Ruminococcus*, etc.

14. **Question:** What microorganisms increase after the addition of probiotics to the feed? **Answer:** *Lactobacillus*, *Bifidobacterium*.

15. **Question:** What microorganisms have a significant impact on pig growth? **Answer:** *Lactobacillus*, *Bifidobacterium*.

16. **Question:** What types of microorganisms dominate in pigs with high feed conversion efficiency? **Answer:** *Lactobacillus*, *Bifidobacterium*, *Methanobrevibacter*, etc.

17. **Question:** What studies have examined the impact of sex on the pig gut microbiome? **Answer:** Studies investigate whether there are differences in pigs of different sexes under various experimental conditions.

18. **Question:** What microorganisms affect pig carcass traits? **Answer:** *Lactobacillus*, *Prevotella*, etc.

19. **Question:** What microbial changes are associated with improved feed efficiency? **Answer:** *Lactobacillus*, *Bifidobacterium*, *Bacteroides*, etc.

20. **Question:** What serum indicators are involved in the studies? **Answer:** High-density lipoprotein, low-density lipoprotein, total cholesterol, triglycerides, etc.

21. **Question:** What indicators are used to evaluate microbial diversity? **Answer:** OTU, Shannon diversity, Simpson index, Alpha diversity, Beta diversity, etc.

22. **Question:** How do feed additives affect the pig microbiota? **Answer:** They can increase or decrease microbial diversity.

23. **Question:** How does pig breed affect the composition of its gut microbiome? **Answer:** Different pig breeds have distinct microbiomes.

24. **Question:** What microorganisms show significant changes under feed additives? **Answer:** *Lactobacillus*, *Bacteroides*, *Prevotella*, etc.

25. **Question:** What is the impact of fermented feed on pig growth performance? **Answer:** increase, decrease.

26. **Question:** What type of microorganisms in the pig gut are most commonly associated with diseases? **Answer:** *E. coli* and diarrhea.

27. **Question:** What is the effect of different feed formulas on pig gut microbiota? **Answer:** increase, decrease.

28. **Question:** What indicators are generally included in the evaluation of feed nutritional value? **Answer:** Dry matter, crude protein, crude fat, crude fiber, nitrogen-free extract, crude ash, etc.

29. **Question:** What are the common sampling sites for gut microbiota? **Answer:** Jejunum, colon, ileum, cecum, duodenum, foregut, hindgut, etc.

30. **Question:** What feed additives are most effective in improving pig gut health? **Answer:** Probiotics, epidermal growth factor.

| **Question 1: What pig breeds are used to study the relationship between the pig gut microbiome and feed efficiency? Answer: Large White, Duroc, Landrace, etc.**  MATCH (n:SwineBreed) RETURN n  n: Duroc × Landrace × Yorkshire, Duroc×(Landrace×Yorkshire), Landrace, Duroc, Large White, Landrace × Yorkshire, Bama mini-piglets, Pietrain, native × Duroc, Jinhua pigs, Jeju native pigs, Meishan gilts, Large White × Créole, Duroc-Danbred × (Landrace × Large White)  **Question 2: At what age stages of pigs have gut microbiome changes been studied? Answer: Piglets, growing-finishing pigs, adult pigs.**  **MATCH (n:SwineStage) RETURN n**  **n: piglets, freshly weaned pigs, neonatal pigs, weanling piglets, finishing pigs, grow‑finishing pigs, nursery pigs**  *^#^There is an incorrect answer: Duroc × (Landrace × Yorkshire), indicating that the annotation is incorrect and needs to be fixed.*  **Question 3: What are the common types of feed additives? Answer: Trace elements, vitamins, amino acids, Chinese herbal medicine, probiotics, etc.**  MATCH (n:FeedadditivesType) RETURN n  n: herbaceous perennial plant, Epidermal growth factor, enzyme supplementation,Compound Probiotics and Berberine, carbohydrases, Organic acids, minerals, prebiotics, glycine, laminarin, etc.  **Question 4: What experimental grouping methods are mentioned in the literature? Answer: Control and experimental groupings, different feed component groupings.**  MATCH (n:ExperimentGroup) RETURN n  n: treatment groups, control group, fed the low-level dose of chlortetracycline, Challenged groups, pigs fed 4000 U/kg or 6000 U/kg AT-xynA, etc.  **Question 5: What fermented feeds are commonly used in experimental designs? Answer: Cassava residue, straw-processed feed, cottonseed meal, fruit residue, distiller's grains, rice bran, etc.**  MATCH (n:FeedFermentationType) RETURN n  n: Live Yeast, cereal fermentation, fermentable carbohydrates, fermented spent mushroom substrates, fermenting dietary polysaccharides, fermented soybean meal, fermented with a newly isolated lactic acid bacteria (LAB) strains combination, fermented liquid feed, etc.  **Question 6: What metabolic pathways might regulate feed efficiency? Answer: Carbohydrate metabolism, amino acid metabolism, glucose metabolism, glycolysis/gluconeogenesis, tricarboxylic acid cycle, cellulose degradation, etc.**  MATCH (n:MetabolismName) RETURN n  n: fiber degradation, tricarboxylic acid cycle, caprolactam degradation, C5-branched dibasic acid metabolism, nucleotide-binding oligomerization-like receptor signaling pathway, limonene and pinene degradation, etc.  **Question 7: What metabolites are involved in the studies? Answer: Butyrate, short-chain fatty acids, benzoic acid, glyoxylate, dicarboxylate, etc.**  MATCH (n:MetabolitesName) RETURN n  n: butyrate, butyric, acetic, lactic acid, SCFAs, propionic acid, 4-aminobutanoate, β-alanine, glycine, 1,3-diaminopropane, creatine, skatole, 5-aminopentanoate, threitol, inosine, polyamine, indole, etc.  **Question 8: What diseases are related to changes in the gut microbiome? Answer: Enteritis, diarrhea.**  MATCH (n:DiseaseType) RETURN n  n: Piglet diarrhea, post-weaning diarrhea, Salmonella infection  *^#^There is an incorrect answer: intestinal epithelium, indicating that the annotation is incorrect and needs to be fixed.*  **Question 9: What carcass traits might be involved in the studies? Answer: Carcass weight, slaughter rate, carcass length, backfat thickness, loin eye area, lean meat percentage, fat percentage, etc.**  MATCH (n:CarcassTraits) RETURN n  n: backfat thickness, slaughter weight of gilts, intestinal muscle layer thickness, gastrointestinal tract weight, villus height, villus height:crypt depth ratio, crypt depth, ileal goblet cell density, apoptosis rates, etc.  *^#^There are wrong answers. Some indicators, such as villus height, villus height:crypt depth ratio, crypt depth, should be classified as intestinal traits and need to be corrected.*  **Question 10: What are the evaluation indicators of pig gut health? Answer: Villus height, crypt depth, villus height to crypt depth ratio, intestinal cells, etc.**  *^#^This knowledge graph cannot find the answer to this question because indicators such as villus height, villus height:crypt depth ratio, crypt depth, ileal goblet cell density, and apoptosis rates are classified under CarcassTraits. This needs to be corrected.*  **Question 11: What are the commonly used antibiotic alternatives? Answer: Probiotics, plant extracts, acidifiers, oligosaccharides, enzyme preparations, lysozyme, bacteriophages, etc.**  MATCH (n:AntibioticSubstituteType) RETURN n  n: organic acids, bacteriophage cocktail, zinc oxide, Lactobacillus plantarum (strains 22F and 25F), Pediococcus acidilactici (strain 72N), Lactobacillus  **Question 12: What genes might be mentioned in the studies? Answer: *TNF-α, TGF-β, IFN-γ, IL-2, IL-1β, IL-6, IL-10, macB*, etc.**  MATCH (n:GeneName) RETURN n  n: *TNF-α, IL-2, ZO-1, macB, IL-1β, Toll-Like Receptor 4, IL-6, ermB, tetM, IL6, OCDN, COX2, TGF-β, IL-8, NOD1, MYD88, IFN-γ, NOD2*, etc.  **Question 13: What microorganisms frequently appear in the studies? Answer: *Lactobacillus*, *Bifidobacterium*, *E. coli*, *Ruminococcus*, etc.**  MATCH (n:MicrobiotaName)-[:belong_to]->(PMID)  RETURN n.name AS MicrobiotaName, COUNT(*) AS MentionCount  ORDER BY MentionCount DESC  LIMIT 10  MicrobiotaName MentionCount  "*Actinobacteria*" 1427  "*Lactobacillus*" 891  "*Firmicutes*" 874  "*Streptococcus*" 839  "*Proteobacteria*" 807  "*Lachnospiraceae*" 803  "*Bacteroidetes*" 803  "*Streptococcaceae*" 793  "*Clostridiales*" 783  "*Prevotella*" 781  **Question 14: What microorganisms increase after the addition of probiotics to the feed? Answer: *Lactobacillus*, *Bifidobacterium*.**  MATCH (fa:FeedadditivesType {name: 'Probiotic'})-[belong_to]->(ed:ExperimentDesign),  (eg:ExperimentGroup)-[:belong_to]->(ed),  (eg)-[:increase]->(m:MicrobiotaName)  RETURN m.name AS Microbe, COUNT(*) AS IncreaseCount  ORDER BY IncreaseCount DESC  no records  *^#^This association does not exist in the current knowledge graph and can be added to the knowledge graph in the future.*  **Question 15: What microorganisms have a significant impact on pig growth? Answer: *Lactobacillus*, *Bifidobacterium*.**  MATCH (n:MicrobiotaName)-[r:significantlyassociated]->(m:GrowthPerformance) RETURN n  n: *Lactobacillus*, *Clostridium*, *Dorea*, *Barnesiella*  **Question 16: What types of microorganisms dominate in pigs with high feed conversion efficiency? Answer: *Lactobacillus*, *Bifidobacterium*, *Methanobrevibacter*, etc.**  MATCH (n:MicrobiotaName)-[r]->(m:SwineType{name:'high FE pigs'}) return n  n: lactic acid bacteria, *Prevotella*  **Question 17: What studies have examined the impact of sex on the pig gut microbiome? Answer: Studies investigate whether there are differences in pigs of different sexes under various experimental conditions.**  MATCH (n:SwineSex)-[:belong_to]->(m:PMID), (l:ExperimentDesign)-[:belong_to]->(m) RETURN n,m,l  n: half male and half female, l: basal diet plus 4% compound probiotics  n: barrows and gilts, l: NC supplemented with BE at 1, 2, and 3 g/kg of feed and with benzoic acid at 5 g/kg  **Question 18: What microorganisms affect pig carcass traits? Answer: *Lactobacillus*, *Prevotella*, etc.**  MATCH (n:MicrobiotaName)-[r]->(m:CarcassTraits) RETURN n,r,m  n: *Desulfovibrionaceae*, *Rikenellaceae*, *Streptococcaceae*, *Lactobacillaceae*, *Prevotellaceae*, *Ruminococcaceae*, *Lachnospiraceae*, m: BFT  **Question 19: What microbial changes are associated with improved feed efficiency? Answer: *Lactobacillus*, *Bifidobacterium*, *Bacteroides*, etc.**  MATCH (n:MicrobiotaName)-[r:increase]->(m:FE) RETURN n  n: *Cellulosilyticum*, *Leeia*, *Subdoligranulu*, *Rothia*, *Lactobacillus*, *Methanobrevibacter*, *Treponema*, *Bacteroidales*, *Clostridiales*, *Lactobacillus johnsonii L531*  **Question 20: What serum indicators are involved in the studies? Answer: High-density lipoprotein, low-density lipoprotein, total cholesterol, triglycerides, etc.**  MATCH (n:SerumIndex) RETURN n  n: serum immunoglobulins G, Serum total protein, serum albumin, Serum globulin, serum urea nitrogen content, high-density lipoproteins, total cholesterol, total triglyceride, serum triiodothyronine, serum tumor necrosis factor-α, etc.  **Question 21: What indicators are used to evaluate microbial diversity? Answer: OTU, Shannon diversity, Simpson index, Alpha diversity, Beta diversity, etc.**  MATCH (n:MicrobiotaDiversity) RETURN n  n: Shannon diversity, alpha diversity, richness and evenness, OTU, beta-diversity, Simpson diversity, microbial diversity, etc.  **Question 22: How do feed additives affect the pig microbiota? Answer: They can increase or decrease microbial diversity.**  MATCH (n:FeedadditivesType)-[r]->(m:MicrobiotaDiversity) RETURN n,r,m  n: probiotics, r: increase, m: Species richness, colon bacterial diversity, alpha diversity index  **Question 23: How does pig breed affect the composition of its gut microbiome? Answer: Different pig breeds have distinct microbiomes.**  MATCH (n:SwineBreed)-[r]->(m:MicrobiotaName) RETURN n,r,m  n: Landrace, r: significantlyhigher, m: *Bacteroides*  n: Duroc, r: significantlyhigher, m: *Clostridium*, *Catenibacterium*  n: Bama mini-pigs, r: shigher, m: *Pseudomonas aeruginosa*, *Escherichia coli*, *Prevotella*, *Faecalibacterium prausnitzii*, *Clostridium coccoides–Eubacteria rectal*, *Bifidobacterium*, *Lactobacillus*  **Question 24: What microorganisms show significant changes under feed additives? Answer: *Lactobacillus*, *Bacteroides*, *Prevotella*, etc.**  MATCH (n:FeedadditivesType)-[r:downregulate]->(m:MicrobiotaName) RETURN n,m  n: CPB, m: *Protebactreria*, *Spirochaetae*  **Question 25: What type of microorganisms in the pig gut are most commonly associated with diseases? Answer: *E. coli* and diarrhea.**  MATCH (n:FeedFermentationType)-[r]->(m:GrowthPerformance) RETURN n,r,m  n: Fermented soybean meal, r: increase, m: ADFI, average daily gain, body weight  **Question 26: What type of microorganisms in the pig gut are most commonly associated with diseases? Answer: *E. coli* and diarrhea.**  MATCH (n:MicrobiotaName)-[r:correlatedwith]->(m:DiseaseType) RETURN n,m  n: *B. licheniformis SDZD02*, *B. amyloliquefaciens DN6502*, *Bacillus subtilis Y-15* m: Piglet diarrhea  **Question 27: What is the effect of different feed formulas on pig gut microbiota? Answer: increase, decrease.**  MATCH (n:DietType)-[r]->(m:MicrobiotaName) RETURN n,r,m  n: wheat bran fiber, r: increase, m: *Bifidobacterium*  n: soybean fiber, r: increase, m: *Escherichia coli*  n: soybean fiber, r: decrease, m: *Lactobacillus* **Question 28: What indicators are generally included in the evaluation of feed nutritional value? Answer: Dry matter, crude protein, crude fat, crude fiber, nitrogen-free extract, crude ash, etc.**  MATCH (n:NutrientType) RETURN n  n: dry matter, crude protein, crude ash  **Question 29: What are the common sampling sites for gut microbiota? Answer: Jejunum, colon, ileum, cecum, duodenum, foregut, hindgut, etc.**  MATCH (n:SamplingSites) RETURN n  n: cecum, Ileum, jejunum, large intestine, hindgut, small intestine, duodenum, rectum, etc.  **Question 30: What feed additives are most effective in improving pig gut health? Answer: Probiotics, epidermal growth factor.**  MATCH (n:FeedadditivesType)-[r:increase]->(m:GrowthPerformance) RETURN n,m  n: BioPlus YC probiotic (Chr. Hansen), probiotics, EGF, m: average daily gain, weight gain, final body weight |
| --- |
